# An infected urachal cyst presenting as acute abdominal pain in a child: A case report

**DOI:** 10.1097/MD.0000000000018884

**Published:** 2020-01-31

**Authors:** I-Shan Tsai, Lung-Huang Lin, Shih-Pin Hung

**Affiliations:** a Department of Pediatrics, Fu Jen Catholic University Hospital, Fu Jen Catholic University, New Taipei City; b Department of Pediatrics, Cathay General Hospital, Taipei, Taiwan.

**Keywords:** abdominal pain, case report, infected urachal cyst, urachal anomalies, urachal cyst, urachal remnants

## Abstract

**Introduction:**

Urachal cyst is an exceptionally rare disease in children caused by the incomplete obliteration of the urachal remnant. Urachal cysts seldom cause symptoms unless a secondary infection occurs. The symptoms of an infected urachal cyst are nonspecific and may be similar to acute appendicitis or other acute abdominal conditions. However, complications attributable to a delayed diagnosis can endanger the life of a patient.

**Patient concerns:**

A 5-year-old boy presented with a 3-day history of severe intermittent lower abdominal pain.

**Diagnosis:**

Infected urachal cyst.

**Interventions:**

The patient was treated with surgical resection of the urachus, followed by intravenous antibiotics during the hospitalization.

**Outcomes:**

The patient was discharged without incident 7 days after the operation. With his follow-up in our out-patient department, he recovered well without any sequelae in the 6 months post-surgery.

**Conclusion:**

We suggested using the abdominal echo scan to differentiate the urachal cyst because of its high sensitivity and nonradioactive characteristic, and computed tomography is a typical diagnostic tool for urachal cysts. The mainstream management of an infected urachal cyst remains surgical excision. Complete excision of urachal cysts is relatively easy in a pediatric patient and the risk of subsequent infection is low; however, patients tend to have a low, although possible, risk of potential malignant transformation over their lifetimes.

## 1 Introduction

The urachus, which connects to the urinary bladder, is a tubular embryonic structure derived from the allantois. Generally, the urachus is obliterated and forms a fibrous core before birth; however, some studies have suggested that this process may occur postnatally.^[1,2]^ Urachal patency results from the failure of the obliterative embryologic process. A urachal cyst is a congenital urachal anomaly attributable to the incomplete regression of the urachus during development in which both ends of the canal close. The incidence of urachal anomalies is thought to be rare. Asymptomatic urachal remnants may be present in up to 2% of the general population according to autopsy studies, but they are rarely symptomatic.^[3]^ Most urachal cysts are asymptomatic unless an infection occurs.^[4]^ In this paper, we report the case of a 9-year-old boy who received a diagnosis of an infected urachal cyst.

Surgical management for urachal cysts used to be the standard of care. However, there has been more recommendation for observation and regular follow-up for urachal cyst in recent 1 decade. For an infected cyst in children, the necessity of surgical management is still the subject of debate. In this case, the patient received excision surgery and recovered smoothly.

This case report was approved by the Institutional Review Board of Cathay General Hospital, Taipei, Taiwan. Informed consent was obtained from the patient and his parents.

## 2 Case presentation

**Figure 1:**
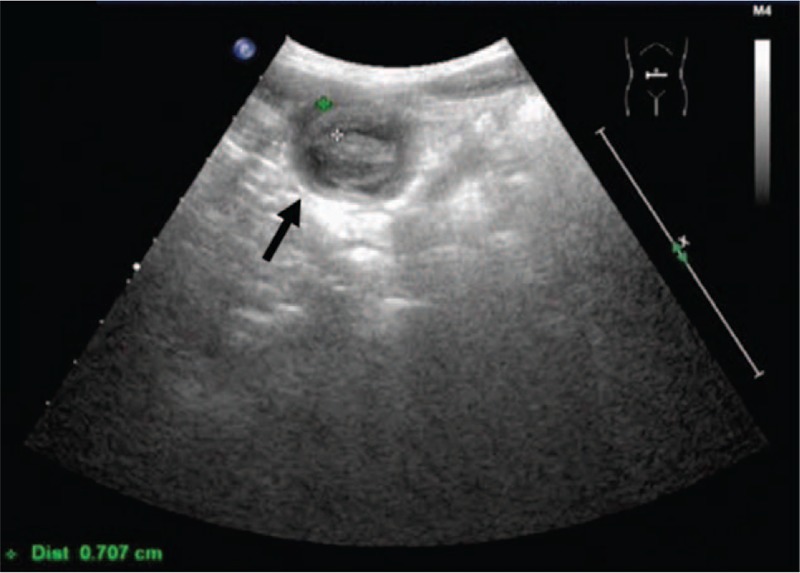
Transverse view of abdominal echography: 1.9 cm hypoechoic mass (black arrow) beneath the midline of the abdominal wall at the dome of the bladder.

**Figure 2:**
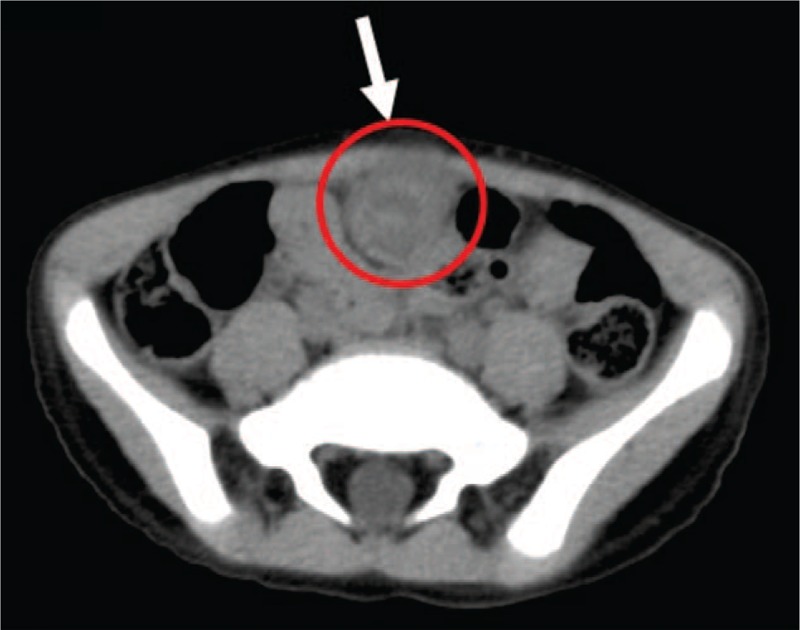
Transverse view of abdominal CT (non-contrast): 2 × 2 cm cystic-like mass lesion (arrow) extending from the upper urinary bladder to the umbilicus with peripheral fat stranding and fluid inside, which suggested an infected urachal cyst. CT = computed tomography.

**Figure 3:**
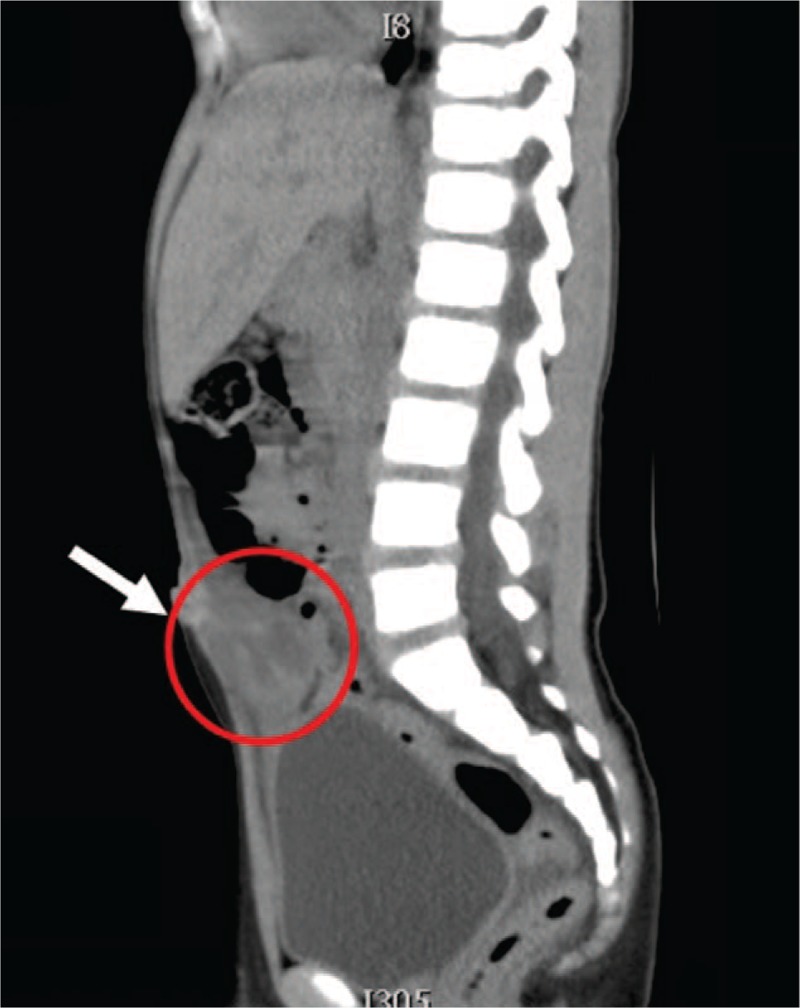
Sagittal and coronal views of abdominal CT (non-contrast): the cystic-like mass lesions located at the apex of the bladder; heterogeneously enhanced cyst inclusion (arrow). CT = computed tomography.

**Figure 4:**
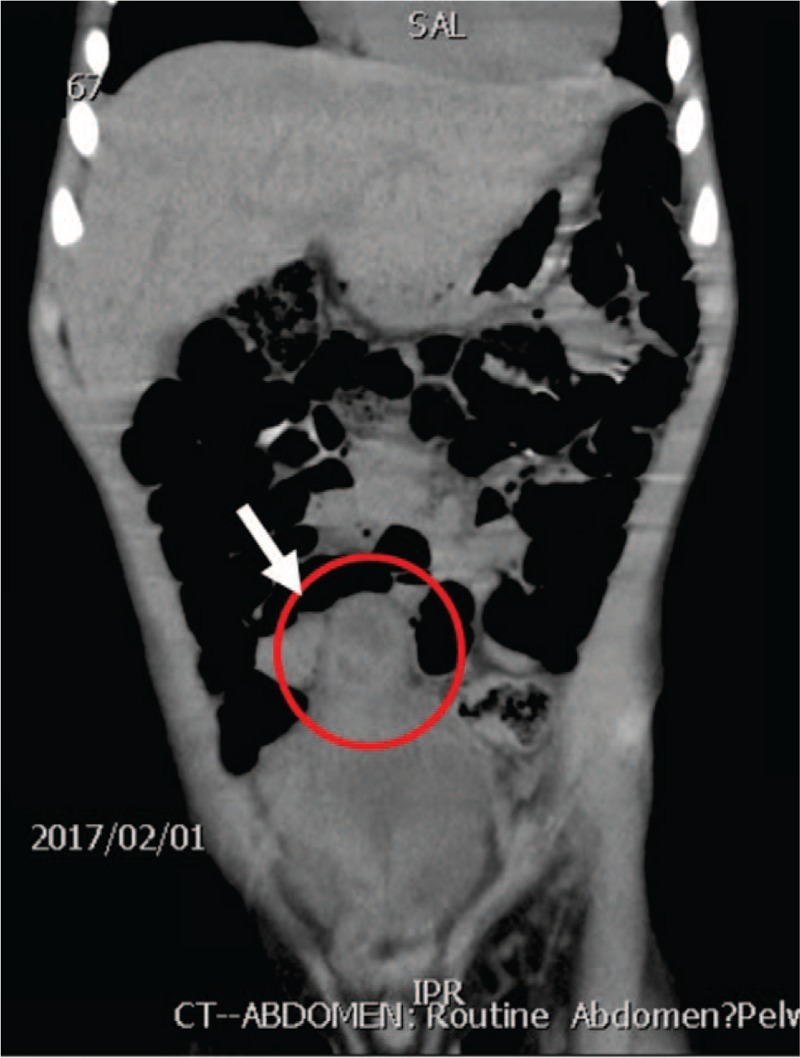
Sagittal and coronal views of abdominal CT (non-contrast): the cystic-like mass lesions located at the apex of the bladder; heterogeneously enhanced cyst inclusion (arrow). CT = computed tomography.

**Figure 5:**
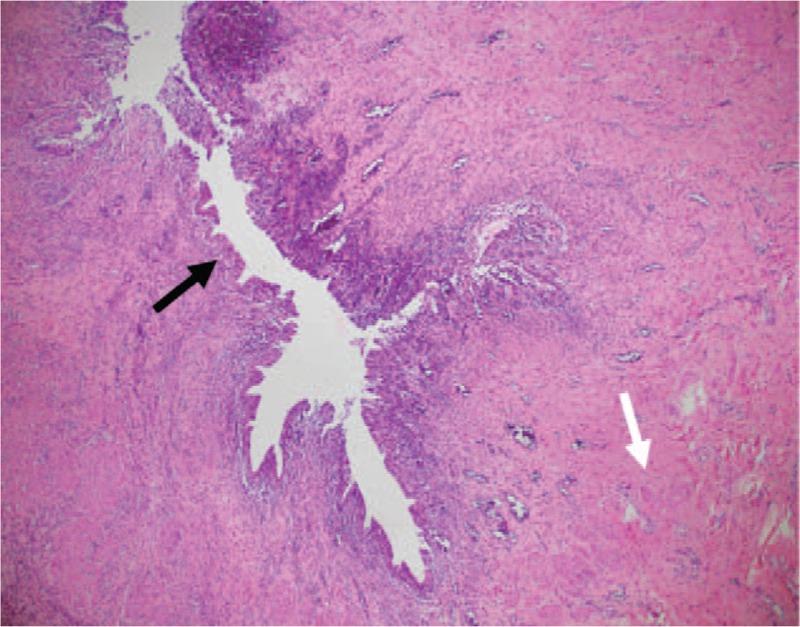
Microscopic view of biopsy specimen taken from the urachal cyst (low power field; hematoxylin and eosin staining): depicts the urothelium (black arrow) and bladder-wall-like tissues (white arrow) identified as having mixed acute and chronic inflammations.

**Figure 6:**
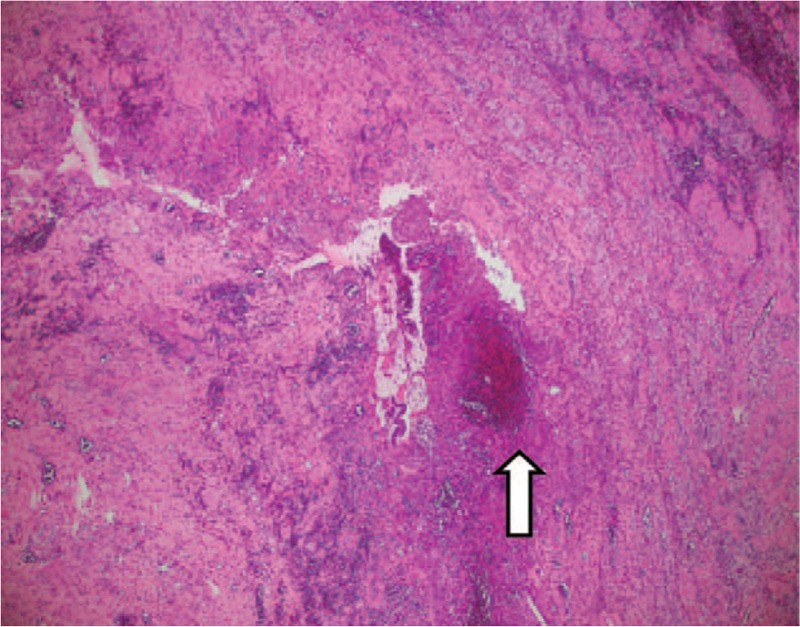
Microscopic view of biopsy specimen taken from the urachal cyst (low power field; hematoxylin and eosin staining): depicts the prominence of the inflamed granulation tissue and focal abscess formation (hollow arrow).

A 5-year-old boy presented with a 3-day history of severe intermittent lower abdominal pain, especially during urination. The patient had been quite healthy without any known underlying medical problem. The pain was exacerbated by standing and urinating and relieved by sitting. He did not complain of nausea and did not experience vomiting or an altered bowel routine. Moreover, he did not have fever, an excessive urinary frequency, a burning sensation while urinating, a skin rash, or purpura. He exhibited a fair degree of activity but a decreased appetite. He was initially brought to an emergency room at hospital A (January 31, 2017). A plain abdominal X-ray revealed stool impaction, and a rectal enema was administered. The urine analysis results were normal. However, the symptoms persisted after he returned home. He was then brought to hospital B for further evaluation and care the next day (January 31, 2017). Urine analysis was performed again, results were normal, and probiotics were administered. Because of continuing abdominal pain, he was subsequently brought to our emergency room 2 days later (February 1, 2017). Examination revealed periumbilical tenderness and a fatigued general appearance, but the condition of the patient was otherwise normal. His body temperature was 36.4°C. Lab results indicated an elevated leukocyte count (13 380/mm^3^) and C-reactive protein level (0.569 mg/dL). Other blood test results were in the normal range. Abdominal ultrasonography (US) revealed a 1.9 cm hypoechoic mass beneath the midline of the abdominal wall at the dome of the bladder (Fig. 1). Intussuception was considered but determined to be unlikely, and an infected urachal cyst was suspected. Computed tomography of the abdomen discerned a nearly 2 × 2 cm cystic-like mass lesion extending from the upper urinary bladder to the umbilicus with peripheral fat stranding and fluid inside, which suggested an infected urachal cyst (Figs. 2–4). Surgical excision of the urachus was performed. During the operation, a residual urachal cyst of more than 5 × 5 cm, adhering to the urinary tract and bladder, was identified. The pathology report described mixed acute and chronic inflammation of the urachal remnant. An inflamed granulation tissue and focal abscess formation were also noted (Figs. 5 and 6). After the operation, the patient's fever rose to 38°C but then subsided spontaneously. Antibiotics were prescribed following the operation, and the patient was discharged without incident 7 days after the operation. The pus culture of the urachal cyst revealed the presence of *Escherichia coli*. With his follow-up in our out-patient department, he recovered well without any sequelae in the 6 months post-surgery.

## 3 Discussion

Urachal anomalies are related to the failure of the developing urachus to completely obliterate its lumen. Urachal anomalies can be diagnosed at any age, and their natural course and predisposing factors are still unknown. Although the actual incidence is unknown, urachal anomalies are thought to be rare. Urachal cyst is one of the congenital urachal anomalies attributable to the incomplete regression of the urachus during development where both ends of the canal close, most commonly at the lower third of the urachal tract. Urachal anomalies may close normally after birth but then reopen in association with pathologic conditions that are often categorized as acquired diseases.^[5]^ The incidence rate of each anomaly has varied among studies. Urachal cysts account for 30% of all urachal anomalies and are the second most common anomaly to patent urachus (50%).^[6]^ In some studies, urachal cysts have been determined to be the most common urachal anomaly, even more frequent than patent urachus in children,^[7–10]^ Urachal cysts were found in 1:5000 autopsies and occurred at a 3:1 male-to-female ratio.^[11]^ In another study, the ratio of boys to girls was 1.2:1 and of men to women was 2.0:1.^[8]^ Vincenzo et al, however, arrived at the opposite finding, concluding that a urachal cyst was the most common in girls (9 of 12; 75%).^[10]^

Most patients with urachal anomalies, except those with a patent urachus, are asymptomatic. Symptoms often accompany a secondary infection, which leads to early discovery.^[12,13]^ Urachal cysts present a variety of clinical manifestations that include localized periumbilical or lower abdominal pain, fever, omphalitis, urinary symptoms, or a painful and palpable mass.^[1,6,8,13]^ Abdominal pain is the most common symptom, according to most studies. The acute abdominal symptoms can be indistinguishable from those of acute appendicitis, Meckel diverticulitis, or peritoneal irritation after perforation of a viscus into the peritoneal cavity.^[14]^ However, a urachal cyst may sometimes occur as an abdominal mass or be noted unexpectedly.^[9]^ Umbilical discharge has sometimes been noted, most typically in infant patients.

To diagnose urachal cyst, a thorough medical history and physical examination are crucial. A definitive diagnosis often requires the use of computed tomography (CT). However, US is the first-line diagnostic tool and is thought to be effective especially for children.^[5]^ Urachal remnants are frequently diagnosed because ultrasound examination is often used as the screening tool. CT or US may reveal a fluid-filled cavity in the midline lower abdominal wall. Once an infection is present, the US image can reveal the complex echogenicity, and the CT image can reveal inhomogeneous attenuation using variable contrast enhancement in and around the disease process. Calcification has seldom been noted in urachal cysts but can be common in urachal malignancies.^[15]^ Some patients receive confirmed diagnoses of a urachal cyst after surgical exploration.^[9]^ A high risk of other related genitourinary anomalies may occur in relation to the urachal remnant.^[10]^ Retrograde urethrography or voiding cystourethrography are used for characterizing urinary bladder patency.

Complications of urachal cysts are often related to infection. If unrecognized, the infected cyst can perforate into the bladder or peritoneal cavity. This can cause peritonitis and the formation of an enteric fistula,^[6]^ and is usually caused by the migration of bacteria, particularly *Staphylococcus aureus*, from the umbilicus.^[13]^ In our case, the pus culture taken from the infected urachal cyst contained *E coli*, which may have had a colorectal origin, and is commonly present in the urachal cyst fluid. Malignancy in adulthood is a concern. Malignancy in a pediatric patient is very rare.^[8]^ The first report of a mucinous urachal neoplasm in a urachal cyst in a pediatric patient was published in 2014.^[15]^ Since then, studies have reported a high rate of malignancies, contrary to the conventional view. Adenocarcinomas related to the urachal remnant remain uncommon, and most patients with simple and asymptomatic lesions do not appear to benefit from prophylactic excision.^[16]^

Surgical management for urachal cysts used to be the standard of care because it could prevent secondary infection and potentially malignant changes later in life.^[4,8,17,18]^ However, spontaneous resolution with nonsurgical management is likely with urachal cysts (and other urachal remnants) in asymptomatic patients younger than 6 month-old.^[10]^ Observation remains a feasible management strategy for asymptomatic young patients because the accessibility of modern US enables a comprehensive follow-up strategy for the first 6 month to year of life. Early intervention (<6 month) should be reserved for patients with documented urine draining from the urachus or a documented abscess.^[19]^

The necessity of surgical management for children with infected urachal cysts is still the subject of debate. Intravenous antibiotic treatment is mandatory for an infected urachal cyst. A review of the literature indicates a 2-stage approach: drainage and antibiotics first and then excision.^[1,20]^ Since 2008, some studies have suggested nonsurgical management, even for infected urachal cysts. Initial drainage of the infected urachal cyst and/or combined antibiotic use with subsequent echography follow-up is sufficient. A study reported that an infected urachal cyst resolved spontaneously.^[21]^ Another study suggested that continuous observation with periodic ultrasound examinations was not necessary for asymptomatic cases because the urachal remnant disappears with age.^[22]^ Long-term follow-up into adulthood in a large patient cohort is necessary to effectively evaluate the necessity of removing the urachal cyst or a urachal remnant.

Most of the existing studies have discussed urachal remnants (or urachal anomalies) other than urachal cysts separately. The clinical manifestation of urachal cysts is often nonspecific and diverse as in our case where the patient complained of acute abdominal symptoms but was misdiagnosed at the initial 2 clinical visits. It can be difficult to make a definite diagnosis through physical examination and medical history evaluation. Imaging methods, such as US and CT, are required. We should also be mindful of the differential diagnosis. In our review of the literature, the optimal management of an infected urachal cyst remained inconclusive. Because the spontaneous regression rate of urachal cysts in older children is not as high as in infants, carcinogenesis can be a risk, secondary infection is a possibility, and pediatric urachal excision can be completed with relative ease, we suggest that a complete urachal excision remains the best choice.

## Author contributions

**Resources:** Lung-Huang Lin, Shih-Pin Hung.

**Supervision:** Lung-Huang Lin, Shih-Pin Hung.

**Writing – original draft:** I Shan Tsai.

**Writing – review & editing:** I Shan Tsai, Lung-Huang Lin, Shih-Pin Hung.
